# The Interplay of Emotional Intelligence Abilities and Work Engagement on Job and Life Satisfaction: Which Emotional Abilities Matter Most for Secondary-School Teachers?

**DOI:** 10.3389/fpsyg.2020.563634

**Published:** 2020-10-20

**Authors:** Sergio Mérida-López, Natalio Extremera

**Affiliations:** Department of Social Psychology, Social Work, Social Anthropology and East Asian Studies, University of Málaga, Málaga, Spain

**Keywords:** work engagement, emotional intelligence, job satisfaction, life satisfaction, interaction, secondary-school teachers

## Abstract

Emotional intelligence has been underscored as a helpful personal resource in explaining life and job attitudes in human services employees. However, the joint interaction of emotional intelligence (EI) abilities with work engagement to explain life and job attitudes has not been tested. The present study aimed to explore the interactive role of EI abilities with work engagement in the prediction of job and life satisfaction in a sample of Spanish secondary-school teachers. A total of 190 teachers (125 females) participated in the study. Notably, the results showed that only emotion regulation ability (ERA) was significantly associated with work engagement, job satisfaction, and life satisfaction. Furthermore, ERA moderated the relationship between work engagement and job and life satisfaction. The present findings contribute to current knowledge on EI abilities and personal and job-related correlates of teachers’ work engagement.

## Introduction

The rise of Positive Organizational Psychology has led to a greater focus on work-related occupational well-being constructs such as work engagement (Mills et al., 2013; Di Fabio, 2017). Work engagement—defined as a positive, fulfilling, work-related state of mind characterized by vigor, dedication, and absorption (Schaufeli et al., 2002)—has received a great deal of attention by both scholars and practitioners worldwide (Bakker et al., 2014). There is an extensive meta-analysis literature on the correlates of work engagement underlying the potential value of this construct for both employees and organizations (Bakker et al., 2014; Knight et al., 2017). In the teaching field, existing studies indicate that work engagement predicts relevant outcomes such as teacher efficacy, satisfaction, and well-being (for a recent overview, see Granziera et al., 2021).

A study with beginning teachers has demonstrated that highly engaged individuals invest more efforts in achieving their work-related goals and, thus, they perform better than their counterparts who experience lower engagement (Bakker and Bal, 2010). When teachers feel vigorous and dedicated they are more satisfied with their job and feel more positive emotions, thereby showing more positive appraisals of their own lives (Upadyaya et al., 2016; Li et al., 2017; Granziera et al., 2021). These latter findings become particularly relevant given that teaching is an occupation at elevated risk for psychological distress (Taris et al., 2017; Harmsen et al., 2018). Despite the considerable amount of research showing the effects of work engagement on personal and work-related well-being, increasing our knowledge on the potential moderating factors in these associations is needed. This research aims to contribute to increase the current knowledge on teachers’ well-being by examining the potential moderating role of emotional intelligence (EI) in the associations between work engagement and teachers’ personal (i.e., life satisfaction) and work-related (i.e., job satisfaction) well-being.

### Teachers’ Work Engagement: Personal and Work-Related Correlates

As stated above, work engagement increasingly represents a critical job-related motivational construct as it relates to several relevant outcomes within educational settings (Taris et al., 2017; Granziera et al., 2021). For example, work engagement is positively associated with teachers’ job satisfaction, which in turn impacts students’ development and academic achievement (Perera et al., 2018). Furthermore, teachers’ work engagement is linked to health and economic benefits associated with reduced absence or high efficacy (Bakker and Bal, 2010; Taris et al., 2017). Since work engagement affects teachers’ personal and work-related well-being, studies on the link between work engagement and individual and work-related correlates of engagement are needed to improve teachers’ work-related quality of life and efficacy (Upadyaya et al., 2016; Perera et al., 2018).

Regarding individual well-being, there is a wide range of affective well-being indicators partially dependent on individuals’ levels of work engagement (Schaufeli and Taris, 2014). For instance, work engagement is associated with higher levels of subjective well-being as well as better physical and mental health (Saks, 2006; Hakanen and Schaufeli, 2012). The Job Demands-Resources (JD-R) theory aligns with the Conservation of Resources (COR) theory (Hobfoll, 2001) illustrating the possible spill-over effects from work to other life spheres. A research with elementary and primary teachers reported a positive association between work engagement and life satisfaction (Pena et al., 2012). Longitudinal evidence has also supported the view that engaged employees generalize their job well-being to their private lives, which is in line with empirical evidence on the causal link between work engagement and life satisfaction (Hakanen and Schaufeli, 2012; Upadyaya et al., 2016). Overall, these results suggest that feeling engaged at work may help fulfilling physical, psychological, and social needs that may positively influence global evaluations of people’s quality of life.

Regarding work-related well-being, earlier research reported significant associations between work engagement with organizational commitment or job satisfaction (Saks, 2006; Schaufeli and Taris, 2014). In line with COR theory (Hobfoll, 2001), teachers with low engagement might feel less attached to their organizations, which may influence their levels of job satisfaction (Sonnentag et al., 2008). Prior research has reported a positive association between work engagement and teacher satisfaction among practicing teachers (Li et al., 2017; Granziera and Perera, 2019). Teachers who are engaged experience positive emotional states that may facilitate more positive affective responses to their work. Conversely, one might expect that teachers with low levels of work engagement would experience reduced work-related well-being (i.e., job satisfaction).

Current knowledge suggest that levels of work engagement are closely related to teachers’ attitudes toward their work and life, which has key implications in educational settings (Perera et al., 2018; Granziera et al., 2021). Although work engagement might be an important factor linked to teachers’ personal and work-related well-being (Schaufeli and Taris, 2014), it is shown that low work engagement does not necessarily lead to low levels of work-related indicators (Parker and Griffin, 2011). Previous research has not paid much attention to the question of how these positive outcomes related to work engagement might be moderated (i.e., to the factors that might facilitate the influence of work engagement on life and job satisfaction). In this study, we examined a personal resource that has attracted increasing attention, namely EI.

### Emotional Intelligence Abilities as Moderators

Increasing research has focused on the impact of EI abilities for teaching (Vesely et al., 2013). In the scientific literature, EI is typically viewed as either an “ability” similar to cognitive intelligence involving cognitive processing of emotional information (Mayer et al., 2016) or an “enduring trait” involving a constellation of emotional self-perceptions located at the lower levels of personality hierarchies (Petrides et al., 2016). The former approach was used in this study. Mayer and Salovey (1997) conceptualized EI as the ability to correctly perceive, facilitate, understand, and regulate emotions to promote personal growth. There is a bulk of research examining the association between EI abilities and individual and work-related well-being domains such as job and life satisfaction (Sánchez-Álvarez et al., 2016; Miao et al., 2017). Emotion regulation ability (ERA) is the most important EI dimension associated with both positive life and job-related attitudes (Joseph and Newman, 2010; Fernández-Berrocal and Extremera, 2016). Thus, the EI construct has gained increased attention with respect to teachers’ work and personal domains (Vesely-Maillefer and Saklofske, 2018; Mérida-López et al., 2019).

The JD-R theory is primarily focused on the role of personal resources (e.g., self-efficacy, optimism, and emotional competences) either as individual predictors of work engagement or as moderators in the relationship between job demands and health outcomes (e.g., Schaufeli and Taris, 2014; Granziera et al., 2021). However, there are theoretical and empirical reasons to expect that EI may moderate the effects of work engagement on employees’ well-being. Considering the facilitating role of EI regarding work-related criteria, it is possible that the presence of certain personal resources such as EI might modulate the association between work engagement and employees’ life and job attitudes (Côté, 2014). Arising from interest concerning the possibility of moderating factors in the consequences of work engagement, there is evidence suggesting that EI may moderate the associations between work engagement and life/job satisfaction. For instance, De Clercq et al. (2014) reported that EI moderated the association between work engagement and organizational deviance. Specifically, employees scoring low in both engagement and EI showed the highest scores in organizational deviance. This aligns with a recent study in which EI acted as a protective factor against teacher turnover (Mérida-López et al., 2020). In this work, those teachers experiencing low engagement, and scoring low in self-report ability EI reported higher turnover intentions than their counterparts with high EI levels. Although the precise place that personal resources such as EI should take within the JD-R theory still remains unclear (Schaufeli and Taris, 2014), these findings shed some light on the importance of considering EI skills as potential moderating factors in the associations between work engagement and its correlates.

Despite growing research focusing on the examination of antecedents and boosting factors of work engagement (Bakker et al., 2014), to date no study has examined whether EI abilities would increase or diminish the influence of experiencing one’s job as engaging on teachers’ job and life attitudes. As such, there is a need to delve deeper into the interplay of work engagement and EI to explain levels of individual and work-related outcomes. Therefore, this study aims to gain insight into the interactive effects of teachers’ work engagement and EI abilities for explaining life and job satisfaction. Based upon past empirical research and the current knowledge on the moderating role of EI (e.g., Côté, 2014; Mérida-López et al., 2020), our expectation is that levels of teachers’ work engagement will be linked to life and job satisfaction, with the strength of these relationships depending on teachers’ EI abilities. As such, it is expected that teachers reporting low levels of engagement do not necessarily exhibit low levels of life and job satisfaction, with this relationship being moderated by available personal resources such as EI abilities. Thus, we hypothesized:

Hypothesis 1: The association between work engagement and life satisfaction (H1a) will be moderated by EI. Similarly, the association between work engagement and job satisfaction (H1b) will be moderated by EI.

## Purpose of the Study

Based on previously mentioned studies and current knowledge on EI, work engagement, and well-being among teachers, the present quantitative cross-sectional study aims to contribute to the literature in three ways. First, it extends the application of COR theory (Hobfoll, 2001), Job-Demands Resources theory (Bakker and Demerouti, 2017), and the moderator model of EI (Côté, 2014) to the educational context. As such, it aims to provide novel evidence on the correlates of work engagement among teachers. Second, this study goes one step beyond previous works that have primarily used self-report instruments to measure ability EI (Miao et al., 2017). Although self-report measures are generally shorter and easier to be used in field studies than performance-based instruments, employing self-report tests may lead to potential inflated statistical relationships between constructs due to common source biases. This study includes a performance-based measure of EI that addresses some of the serious limitations of self-report tests in the EI field (Mayer et al., 2016). Moreover, it follows recommendations in the occupational health psychology field regarding the use of objective measures for assessing resources (Bakker and Demerouti, 2017). It thus may help researchers to gain more insight into the associations among ability EI, work engagement, and job and life satisfaction (Sánchez-Álvarez et al., 2016; Miao et al., 2017). Third, this research expands previous studies as it positions teachers’ work engagement as a motivational construct linked to work-related and general outcomes (Schaufeli and Taris, 2014). In other words, this work is found among the first attempts to test whether employees’ EI might influence the extent to which work engagement relates to their work-related and personal well-being. Given the impact of teaching-related demands (Taris et al., 2017; Granziera et al., 2021), findings from this study might help to develop personal interventions to increase life and job satisfaction among teachers.

## Materials and Methods

### Participants

The study sample was comprised of 220 secondary-school teachers working in different centers of Southern Spain. Thirty participants did not complete the battery, which led to a final sample of 190 teachers (65.8% female) with a mean age of 45.38 years (SD = 8.03, range = 25−63 years). The marital status of the participants was: 56.8% married, 20.5% single, 11.1% separated/divorced, 9.5% couple, and 2.1% unspecified. Average teaching experience was around 17 years (*M* = 17.06 years, SD = 8.74), whereas organizational tenure was around 7 years (*M* = 6.51 years, SD = 6.28). Regarding their educational level, 86.3% of the participants held a 5-year degree, 8.4% held a 3-year degree, and a 3.2% held a doctorate.

### Procedure

A battery of questionnaires was given to teachers through the assistance of members of the research staff underlying the anonymous and confidential nature of the data. The research protocol was approved as part of the project PSI2012-38813 by the Research Ethics Committee of the University of Málaga.

### Measures

Along with socio-demographic variables (i.e., age, gender, academic degree, marital status, teaching experience, and organizational tenure), a battery of questionnaires was included with well-validated measures for the main study variables.

### Work Engagement

The Utrecht Work Engagement Scale (UWES; Schaufeli et al., 2002; Spanish version by Salanova et al., 2000) is a 15-item scale aimed at assessing three dimensions of work engagement (i.e., vigor, dedication, and absorption) using a Likert-type scale. We used the overall work engagement score, given our interest in the whole construct (Mérida-López et al., 2019). This scale has shown adequate psychometric properties in Spanish samples (Extremera et al., 2012). Cronbach’s alpha coefficient was 0.91 in our sample.

### Emotional Intelligence

The Mayer-Salovey-Caruso Emotional Intelligence Test (MSCEIT v.2.0; Mayer et al., 2003; Spanish version by Sanchez-Garcia et al., 2016) is a 141-item scale assessing ability EI. The four dimensions of the test (i.e., emotion perception, emotion facilitation, emotion understanding, and emotion regulation) draw on different tasks including different items forms. For instance, respondents are asked to identify the emotions expressed in photographs of people’s faces (emotion perception). Moreover, individuals are required to read a short story about another person and then are asked to determine how effective several courses of action would be to cope with the emotions in the story (emotion regulation). Considering the test’s items heterogeneity, split-half estimates of reliability are the statistic of choice rather than coefficient alphas (Mayer et al., 2003). This instrument has shown adequate psychometric properties in Spanish samples (Sanchez-Garcia et al., 2016). In this study, the test internal consistency reliability (split-half) coefficients for all sub-dimensions were: 0.92 for emotion perception, 0.52 for emotion facilitation, 0.71 for emotion understanding, and 0.72 for emotion regulation. The split-half reliability for the overall score was 0.91.

### Life Satisfaction

The Satisfaction with Life Scale (SWLS; Diener et al., 1985; Spanish version by Vázquez et al., 2013) is a 5-item instrument aimed at assessing global life. The Spanish version has shown adequate psychometric properties (Vázquez et al., 2013). In this study, Cronbach’s alpha coefficient was 0.86.

### Job Satisfaction

Job satisfaction was measured with a 5-item scale (Judge et al., 1998; Spanish version by Extremera et al., 2018) originally based on a job satisfaction index developed by Brayfield and Rothe (1951). This instrument has shown adequate internal consistency in Spanish samples (Extremera et al., 2018). In this study, Cronbach’s alpha was 0.82.

### Statistical Analyses

First, descriptive statistics and internal consistency were calculated for the main study variables. Second, Pearson bivariate correlations were tested to examine the associations among the study variables. Third, to assess the potential moderator effect of ability EI in the relationship between work engagement with job and life satisfaction, two moderator models were tested with the PROCESS macro (Hayes, 2018). Model 1 was tested for each dependent variable (i.e., life satisfaction and job satisfaction) to examine whether the effect on these variables was moderated by levels of ability EI. Bootstrapping with 5,000 re-samples and 95% bias-corrected confidence intervals was used in line with standard guidelines (Hayes, 2018).

## Results

### Descriptive Statistics and Correlations

Table 1 shows descriptive statistics, correlations, and reliability coefficients for the main variables. As shown, the relationship between perception, assimilation, and understanding abilities with the main variables were non-significant. It is noteworthy that ERA was positively and significantly related to work engagement and job and life satisfaction. Similarly, work engagement was positively associated with both life and job satisfaction.

**TABLE 1:** Descriptive statistics and correlations among the study variables.

Variable	*M* (SD)	α	1	2	3	4	5	6	7	8
1. Emotion perception	100.57 (14.21)	0.92								
2. Emotion facilitation	100.05 (12.51)	0.52	0.44**							
3. Emotion understanding	103.58 (11.08)	0.71	0.21**	0.23**						
4. Emotion regulation	109.37 (10.43)	0.72	0.30**	0.45**	0.20**					
5. Overall EI	103.56 (11.45)	0.91	0.81**	0.74**	0.56**	0.63**				
6. Work engagement	4.72 (0.83)	0.91	–0.01	0.04	–0.01	0.25**	0.07			
7. Life satisfaction	4.99 (1.07)	0.86	0.03	0.10	–0.08	0.20**	0.07	0.34**		
8. Job satisfaction	5.41 (0.99)	0.82	0.03	0.08	0.07	0.25**	0.13	0.81**	0.32**	

**p < 0.01.

### Moderation Analyses

To test our interaction hypothesis that ability EI levels moderate the relationship between work engagement and job and life satisfaction, we conducted two separate moderation analyses for each of the dependent variables. The results are displayed in Table 2. Since ERA was the only EI dimension significantly related to the study variables, analyses were conducted with this EI dimension as a moderator. The PROCESS macro in SPSS (Hayes, 2018) was used to run a series of OLS regressions with the centered product term representing the interaction of work engagement and ERA as a predictor of life and job satisfaction. Age and gender were included as covariates.

**TABLE 2:** Tested moderation model with life and job satisfaction as outcomes.

	Coefficient	SE	95% CI
Life satisfaction	*R*^2^ = 0.21, *F*(5,184) = 9.72***		
Constant	5.92***	0.49	4.9642 to 6.878
Gender	0.06	0.15	−0.2320 to 0.0358
Age	−0.02**	0.01	−0.0414 to −0.0067
Work engagement	0.41***	0.09	0.2401 to 0.5848
Emotion regulation ability	0.02**	0.01	0.0051 to 0.0358
Work engagement × emotion regulation ability	0.03**	0.01	0.0105 to 0.0428
Job satisfaction	*R*^2^ = 0.67, *F*(5,184) = 73.40***		
Constant	5.75***	0.29	5.1710 to 6.3301
Gender	0.07	0.09	−0.1096 to 0.2472
Age	−0.01*	0.01	−0.0211 to −0.0001
Work engagement	0.96***	0.05	0.8519 to 1.0606
Emotion regulation ability	0.01	0.01	−0.0017 to 0.0169
Work engagement × emotion regulation ability	0.01*	0.01	0.0001 to 0.0197

SE, standard error; 95% CI, confidence interval with lower and upper limits. *p < 0.05; **p < 0.01; ***p < 0.001.

Regarding life satisfaction, we found that age was a significant socio-demographic variable explaining variance in life satisfaction (*b* = −0.02, *p <* 0.01). Both work engagement (*b* = 0.41, *p <* 0.001) and ERA (*b* = 0.02, *p <* 0.01) were positively related to life satisfaction. Moreover, the interaction term was significant (*b* = 0.03, *p <* 0.01) and explained 4.5% of unique variance beyond the main effects of work engagement and ERA (Δ*R*^2^ = 0.045, *F* = 10.57, *p <* 0.01). The model predicted 21% of the variance in life satisfaction. Thus, results supported H1a.

**FIGURE 1:**
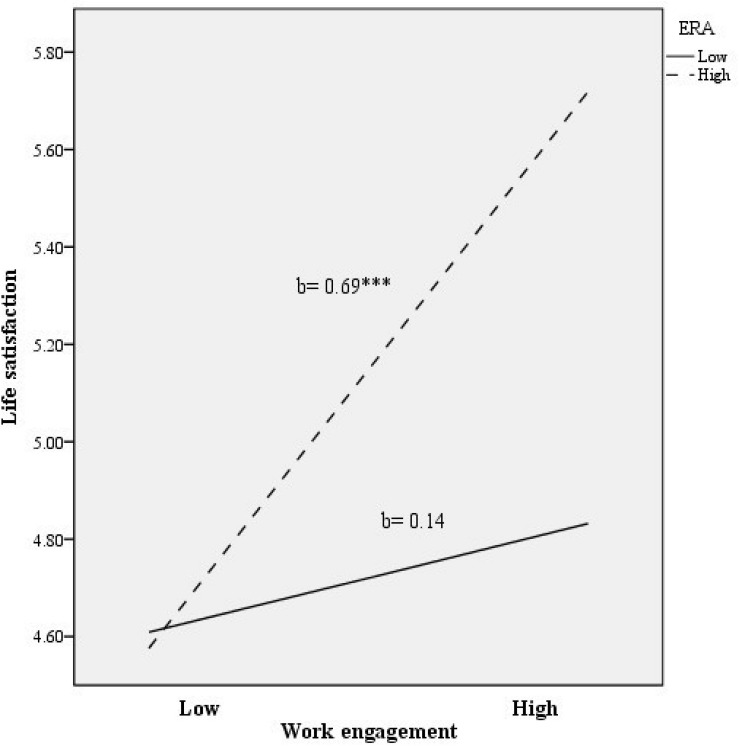
Relationship of work engagement and ERA for predicting life satisfaction. ^∗∗∗^*p* < 0.001.

To illustrate the interaction effect, we followed standard guidelines to examine the relationship between work engagement and life satisfaction regarding low vs. high scores of ERA (i.e., one standard deviation above and below the mean). As shown in Figure 1, the relationship between work engagement and life satisfaction increased when ERA levels were high (vs. low). The positive association between work engagement and life satisfaction was non-significant at low levels of ERA (*b* = 0.14, *t* = 1.11, *p* = 0.27), whereas it became significant at high levels of ERA (*b* = 0.69, *t* = 5.63, *p <* 0.001).

With regard to job satisfaction, the results showed that age was a significant negative predictor (*b* = −0.01, *p <* 0.05). While work engagement was significantly and positively related to job satisfaction (*b* = 0.96, *p <* 0.001), ERA did not show a significant main effect for predicting levels of job satisfaction (*b* = 0.01, *p* = 0.11). However, the interaction term was significant (*b* = 0.01, *p <* 0.05) and accounted for 0.7% of unique variance in job satisfaction beyond the main effects of work engagement (Δ*R*^2^ = 0.007, *F* = 3.97, *p <* 0.05). The model predicted 67% of the variance in job satisfaction. Results provided support for H1b.

**FIGURE 2:**
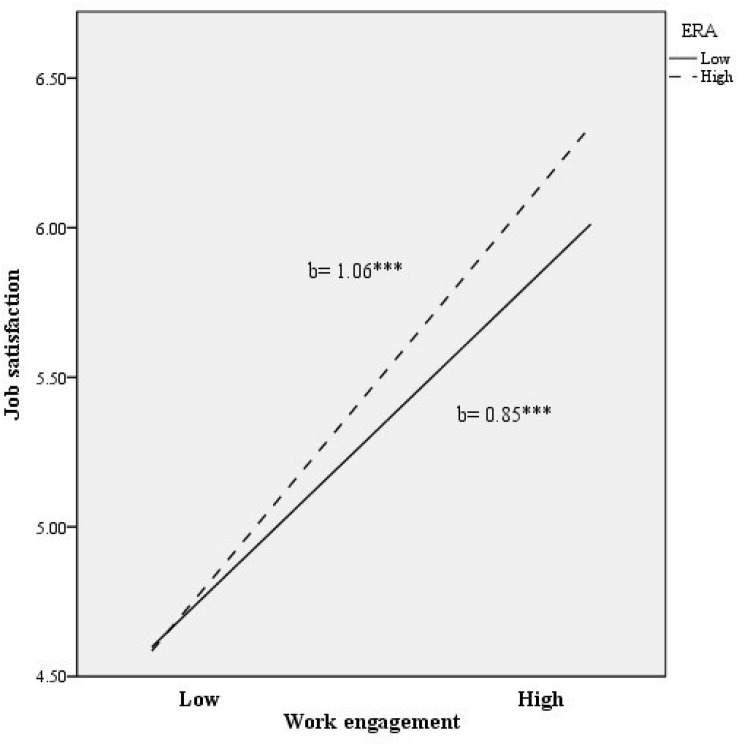
Relationship of work engagement and ERA for predicting job satisfaction. ^∗∗∗^*p* < 0.001.

To illustrate the interaction effect, we examined the relationship between work engagement and job satisfaction regarding low vs. high scores in ERA. As shown in Figure 2, the relationship between work engagement and job satisfaction increased when ERA was higher (vs. lower). Specifically, the positive association between work engagement and job satisfaction was significant at low levels of ERA (*b* = 0.85, *t* = 11.57, *p <* 0.001), whereas it became more intense at high levels of ERA (*b* = 1.06, *t* = 14.29, *p <* 0.001).

## Discussion

Past studies have corroborated a robust link between work engagement levels and positive individual and job-related outcomes in employees (Saks, 2006; Hakanen and Schaufeli, 2012). However, the potential role of personal resources as mechanisms that modulate this relationship remains unclear. To address this gap, this study examined the role of EI abilities as potential moderators in the relationship between work engagement and teachers’ job and life satisfaction.

First, in line with previous studies in teacher samples, our results showed a positive and significant association between work engagement and job satisfaction (Granziera and Perera, 2019). Similarly, the results showed that work engagement was positively associated with life satisfaction, which is consistent with past studies (Pena et al., 2012; Upadyaya et al., 2016). Second, the findings showed ERA as the only component of EI linked to work engagement and job and life satisfaction. These results are in line with previous empirical evidence showing positive associations between ERA and both life (Fernández-Berrocal and Extremera, 2016) and job satisfaction (Brackett et al., 2010; Extremera et al., 2020). Likewise, this accords with prior research with teaching professionals in which ERA was positively associated with teachers’ work engagement (Castillo-Gualda et al., 2017).

The fact that emotion regulation appears to be the solely EI dimension related with work engagement and well-being indicators may be explained in terms of the namely cascading model of EI (Joseph and Newman, 2010). Accordingly, emotion regulation may be a dimension precluding correlates of work engagement such as performance and well-being. Although EI dimensions are critical to help employees to feel committed and satisfied at work so they perform more effectively (Côté, 2014), these findings suggest that the ability to deal with own and others’ emotions might play a key role in promoting well-being and positive emotions between teachers, which could influence a variety of individual and job outcomes among teachers (Mérida-López et al., 2017; Castillo-Gualda et al., 2019). For instance, if a teacher exhibit high levels in emotion regulation they would know that anger is more useful than happiness when confronting a student in an admissible situation. Likewise, when teachers set more adjusted emotion regulation goals and they also implement adaptive emotion management strategies they are more likely to attenuate the potential harmful effects of emotionally demanding situations on their health and motivation at work. In sum, high ERA might improve the way teachers appraise events and situations that might be perceived as a threat in the school, and it might interact with levels of work engagement to cope with handling pressure and stress and positively influence both life and job satisfaction levels.

Regarding the moderating effect of the ability to manage emotions in the relationship between work engagement and life satisfaction, the results showed that the highest levels of life satisfaction were found among teachers reporting high engagement and scoring high (vs. low) in ERA. Thus, although there was a slight increase in life satisfaction among teachers with high engagement, at high vs. low ERA levels, the magnitude was relatively small and non-significant. This pattern is in line with the assumption that life satisfaction is likely to be increased at higher (relative to lower) levels of ERA, and even more in teachers with high engagement. In summary, our findings suggest that although being engaged at work in the teaching profession is associated with life satisfaction, it appears that regulating emotions effectively might matter more in increasing levels of life satisfaction. Low ERA could negatively affect how teachers interpret aspects of their lives, leading to more negative stress reactions and thereby reducing their perception of overall life satisfaction.

Although the association between work engagement and job satisfaction was positive for both low and high levels of ERA, this EI dimension strengthened the relationship between the variables. This result suggests that teachers who are more skilled at shaping their own and others’ emotions and perceiving their work as engaging are more satisfied with their jobs than their counterparts with lower ERA. In line with a recent meta-analytic review on EI and job satisfaction, ERA could be a potential resource through which positive work attitudes of teachers can be boosted (Miao et al., 2017).

It is noteworthy that the results suggest a differential pattern in terms of the different spheres of well-being. While the relationship between work engagement and job satisfaction remained positive for teachers scoring low in ERA, the association between work engagement and life satisfaction was non-significant for teachers scoring low in ERA. These findings could suggest that ERA, when interacting with teachers’ work engagement, exerts a more intense influence when explaining levels of personal rather than job-related attitudes. One potential reason might be that, to some degree, fostering positive attitudes at work (i.e., job satisfaction) might be more dependent on organizational factors—which are external rather than internal—such as school climate, number of students, or perceived school support (Geiger and Pivovarova, 2018). Moreover, our findings showed that the interaction between work engagement and ERA significantly augmented the prediction of both job and life satisfaction. Although the full model for job satisfaction explained a higher proportion of the variance than the life satisfaction model, the interaction term explained more additional variance in the life satisfaction model than in the job satisfaction model. One tentative justification might be that, although conceptually different, work engagement is a closer theoretical construct to job satisfaction—that is, while work engagement is concerned with the employee’s mood at work, job satisfaction is concerned with affect toward work (Schaufeli and Bakker, 2010). Likewise, both work-related constructs show generally higher significant shared common variance, with ERA showing a less relevant role. Pending replication, it is plausible that the joint effect of work engagement and ERA might play a particular role in explaining life and job well-being in employees. Future researchers are advised to examine this issue in depth.

Our study has some limitations. First, due to the cross-sectional nature, causal links among variables should be drawn with caution. Although the proposed hypotheses relied in previous research and in robust current theories on emotions at work and well-being, future studies are advised to replicate the present findings with longitudinal designs (Burić and Macuka, 2018). Despite this study is found among the first empirical approaches to assess the relationship between performance-based ability EI and teachers’ work engagement (Castillo-Gualda et al., 2017), a second limitation relates to the relatively small sample size. Likewise, the data presented in this study represent secondary-school teachers from southern Spain. It should be noted that using a performance-based EI measure is expensive and time-consuming in comparison with a self-report instrument, which may cause practical difficulties in field studies. Moreover, equivalent sample sizes have been considered in prior research using the MSCEIT with teaching professionals (e.g., Brackett et al., 2010; Castillo-Gualda et al., 2019). Unfortunately, these studies solely measured ERA and did not report data on the remaining EI abilities. Such approach merits attention as it could advance understanding on the correlates of performance-based EI dimensions (Côté, 2014; Miao et al., 2017). Therefore, studies with larger and more representative samples are needed to replicate current findings. Future studies are advised to explore how organizational factors and emotional abilities relate to teachers’ well-being (Yin et al., 2016). Third, future work should assess multiple-level factors influencing teachers’ general and work-related well-being (Yang et al., 2019). Moreover, researchers are advised to test the joint contribution of personal resources (e.g., EI dimensions, self-efficacy, and resilience) to well-being and work engagement (Molero Jurado et al., 2019). Finally, researchers should assess specific teaching-related job characteristics and their relationship with work engagement and teachers’ well-being (Taris et al., 2017; Salmela-Aro et al., 2019). In this regard, future studies should profitably use profession-specific measures through which teachers’ knowledge and skills regarding EI key dimensions can be assessed (e.g., situational judgment tests such as the Test of Regulation in and Understanding of Social Situations in Teaching; Aldrup et al., 2020).

### Theoretical and Practical Implications

This study represents a novel contribution to the literature on teachers’ emotions and work-related well-being as it adds to current knowledge on the associations between ability EI and individual and job-related well-being indicators in a sample of secondary-school teachers. Although there is research that has examined interactive effects of job demands and personal resources on work engagement and burnout levels (Xanthopoulou et al., 2007), to date no study has examined the influence of certain personal variables as moderators in later stages of the JD-R theory. Thus, these results are a starting point for future studies contributing to a better understanding of the individual factors modulating the effects of work engagement on general and organizational outcomes (Schaufeli and Taris, 2014).

Given that the JD-R theory continues to be updated such as the inclusion of reciprocal relationships between job resources and work engagement, the applicability to different contexts, and the distinction between hindrance and challenge demands (Bakker et al., 2014; Bakker and Demerouti, 2017), our work makes a significant contribution by providing evidence of personal resources that facilitate both spill-over effects from work engagement to life satisfaction and effects of work engagement on job satisfaction (Côté, 2014; Miao et al., 2017). This study also contributes to the relatively scarce literature on correlates of work engagement regarding personal well-being outcomes in comparison with work-related outcomes (Upadyaya et al., 2016).

With respect to practical implications, our results could help human resources practitioners to assess potential deficits in both work engagement and ERA related to poor job and life satisfaction to identify teachers who could be at risk of developing work-related problems. Following previous and current findings, teacher engagement development would not be sufficient itself to predict the highest levels of life and job satisfaction (Bakker et al., 2014; Taris et al., 2017). Because personal resources such as emotion regulation are capacities that individuals may be able to develop or change, they provide a promising base from which interventions targeting teacher individual and work-related well-being may be developed. In fact, existing results on the benefits of interventions targeting EI to increase well-being among teachers seem promising (Castillo-Gualda et al., 2019; Schoeps et al., 2019). Thus, school practitioners are advised to address individual-organizational interface interventions for improving individual and work-related well-being (Randall and Travers, 2017). For instance, they could implement and develop programs targeted at improving educational practices or contextual characteristics that could enhance engagement at work—for example, allowing teachers to change school-related characteristics causing the most dissatisfaction among staff (i.e., job crafting; Van Wingerden et al., 2017) and increasing personal resources (i.e., emotion regulation strategies) among teachers at risk, reducing or modifying whatever maladaptive regulation strategies they use (e.g., rumination, self-blame) and offering more adaptive ones (e.g., refocus on planning or positive reappraisal) so they can develop and hold more positive attitudes toward their lives and teaching jobs.

Given that work-related stress among teachers conveys a wide range of negative outcomes, investing efforts to improve levels of work engagement and emotional resources among the teaching staff might be important to prevent from work issues with substantial health and economic burdens such as presenteeism, sickness absenteeism, or intention to quit (Taris et al., 2017; Harmsen et al., 2018). In sum, our results suggest new paths in which emotional abilities such as emotion regulation could, in combination with work motivational constructs, contribute to the prediction of teachers’ well-being. Since developing work engagement and fostering emotion regulation abilities is crucial for social and personal functioning in teachers’ lives, the design and implementation of training programs for improving these dimensions appears to be a promising avenue for improving teachers’ quality of work life.

## Data Availability Statement

The datasets presented in this article are not readily available because the dataset has been generated regarding a funded Research Project by Junta de Andalucia/FEDER funds (UMA18-FEDERJA-147). Requests to access the datasets should be directed to NE, nextremera@uma.es.

## Ethics Statement

The studies involving human participants were reviewed and approved by the Research Ethics Committee of the University of Málaga (66-2018-H). The patients/participants provided their written informed consent to participate in this study.

## Author Contributions

Both authors conceived and designed the research, analyzed the data, contributed reagents, materials, and analysis tools, wrote the manuscript, prepared the tables, and reviewed drafts of the manuscript.

## Conflict of Interest

The authors declare that the research was conducted in the absence of any commercial or financial relationships that could be construed as a potential conflict of interest.
